# Menstrual Morbidities, Menstrual Hygiene, Cultural Practices during Menstruation, and WASH Practices at Schools in Adolescent Girls of North Karnataka, India: A Cross-Sectional Prospective Study

**DOI:** 10.1155/2020/6238193

**Published:** 2020-07-16

**Authors:** Rajasri G. Yaliwal, Aruna M. Biradar, Shreedevi S. Kori, Subhashchandra R. Mudanur, Shivakumar U. Pujeri, Mohd Shannawaz

**Affiliations:** ^1^Department of Obstetrics and Gynecology, BLDE (Deemed to be University), Shri B. M. Patil Medical College, Hospital and Research Center, Vijayapura, Karnataka 586103, India; ^2^Department of Community Medicine, BLDE (Deemed to be University), Shri B. M. Patil Medical College, Hospital and Research Center, Vijayapura, Karnataka 586103, India

## Abstract

**Background:**

Issues of menstrual morbidities, menstrual hygiene, and cultural practices are rarely discussed by adolescents. The burden of menstruation and cultural practices which the adolescent girls have to face has been less quantified. This study aims to assess the issues related to menstruation in school girls.

**Method:**

A cross-sectional prospective study was conducted on 1016 school-going adolescent girls in January 2020. A questionnaire in English and in Kannada was distributed to girls of class 8–12 of ages between 10 and 19 years.

**Results:**

70.5% of the girls attained menarche by 12 to 14.9 years, 37.2% of the girls had their periods every 28–34 days, and 12.2% of the girls said they have heavy periods. 61.95% of the girls had dysmenorrheal, and 9.7% of the girls said that they required medications for the pain. 70.7% of the girls were using commercial sanitary napkins, 12.7% were using cloth, and 15.3% were using both. 55.5% of the girls who were using cloth as an absorbent were not drying the cloth in sunlight. 57.1% of the girls were washing their genitals more than 2 times a day. 93.8% were having bath during menses and 87.2% were using soap along with water. 37.7% of the girls disposed their pads by burning them, 50.8% of then disposed them in the dust bin, and 4.9% of them buried them. 8.6% of the girls said that they remained completely absent from school during periods. 17.85% said that they remained absent for a day. 53.4% of the respondents said that they have difficulty in concentrating at school. 76.1% said that they had adequate water and sanitation facilities at school. 22.3% said that there was adequate facility to change their pads at school. 73.2% said that they could get a spare pad at school. 43.3% of the girls said they avoided cultural functions during their periods, and 38.5% said that they avoided religious ceremonies and practices during their periods. 8.7% of the girls were made to sit outside the house during their periods. The girls from rural areas had poorer hygienic habits, in comparison to the urban girls. Cultural restrictions such as sitting outside the house during menstruation and restricting play were more in the rural girls than the urban girls.

**Conclusion:**

Menstrual morbidities, menstrual hygiene management, and cultural beliefs all play a role in school absenteeism in adolescent girls. Improvement of facilities at school and conducting awareness programs can help adolescent girls to attend schools.

## 1. Background

Menstrual hygiene is a subject which is as old as humanity but has gained recent importance due to the readiness of the society to accept its challenges.

Attaining menarche is a celebrated event across cultures. The physiological and psychological changes that the girl endures are also associated with the stress of menstrual hygiene management. Menstrual hygiene management (MHM) at school is very important as well. The WHO (World Health Organization) and UNICEF (United Nations International Children's Emergency Fund) advice WASH facilities at school, i.e. water, sanitation, and hygiene [1]. In India, the Swachh Bharat: Swachh Vidyalaya campaign has been launched in every school to provide WASH facilities, which includes soap and water for sanitation and private space for changing and disposal of menstrual absorbents. MHM has been made an integral part of the Swachh Bharath guidelines. Efforts are being made to provide low-cost sanitary napkin vending machines and incinerators to dispose MHM products at schools [2]. However, the extent to which all these guidelines percolate down to the ground level has yet to be seen. Inadequate facilities at the school may, in turn, result in school absenteeism and diminished school performance. Various absorbents have been used during the menstruation. The reusable absorbents are made up of cloth. They need to be washed and dried in sunlight prior to the next use. The nonreusable sanitary pads are made up of cellulose and plastic. They are user friendly. However, they are expensive and they are nonbiodegradable. Bamboo fiber pad, banana fiber pad, and water hyacinth pad are the biodegradable ecofriendly sanitary napkins. They are not readily available. Reusable and nonreusable tampons are also available. Menstrual cups are also used. These have to be inserted in the vagina. Hence, they are not suitable for all adolescents.

Nonreusable pads have to be disposed in the dustbins. However cultural beliefs and lack of disposal facility have made certain communities burry or burn the pads [3]. Reusable cloths are to be washed with soap and dried in the sunlight to prevent growth of bacteria. Due to cultural beliefs, the cloths are not properly washed with soap, and many a time, they are kept to dry away from the sunlight and away from the sight of other family members. Such unhygienic practices lead to vaginitis, pelvic infections, and urinary tract infections [4, 5].

Minor ailments during the menstrual period are common. Abdominal pain, lack of concentration, and breast pricking are common complaints [6].

These symptoms may require help from the teachers at school. Approachability and sensitivity of the teacher are also very important for the girls.

Menstruation is still considered as a taboo across various cultures, and young girls of some communities feel that that menstruation is a curse or burden [7]. Women in their menstrual period are considered as filthy, shameful, or impure. Many communities restrict menstruating women from various activities such as cooking, touching food, being with family members, attending religious ceremonies, and bathing [8]. Menstruation is a physiological process, and such taboos and myths should be allayed.

The present study aims to study the sociodemographic characteristics of adolescent girls, details regarding the menstrual cycle, menstrual hygiene practices, MHM at schools, and cultural beliefs followed during menstruation. To the best of our knowledge, this is the first time a study on MHM is being conducted in Vijayapura, North Karnataka, India.

All girls from class 8 to 12 who had attained menarche were included in the study. Adolescents are defined as girls from age 10–19 years of age. Girls who were of ages below 10 and above 19 were excluded from the study.

## 2. Materials and Methods

The cross-sectional prospective study was conducted in January 2020. The study was conducted in 10 schools which included girls from class 8 to class 12 in the district of Vijayapura, Karnataka. Vijayapura is situated in North Karnataka which is considered as less developed as in comparison to its southern counterpart. The students attending the schools were of low- and middle-income groups. Girls attending the schools were from the Vijayapura city and the surrounding villages. Ethical clearance was given by the institutional ethical clearance committee, BLDE (deemed to be university), Vijayapura.

Consent from the head of each school was taken before conducting the study. A consent form was given to each student who was involved in the study and was asked to get it signed from their parent. A questionnaire covering the demographic characteristics of the participants, characteristics of the menstrual cycle and menstruation in the participants, menstrual morbidities experienced by the participants, menstrual hygiene practices among the participants, difficulties faced at schools by the participants during menstruation, and cultural beliefs practiced by the participants was prepared in English. As a majority of the schools had Kannada as the medium of instruction, the English questionnaire was translated into Kannada. Kannada is the largest spoken language of the region, and it is also the official language of Karnataka State. The Kannada and English questionnaires were distributed to the girls who were from schools where the medium of instruction was Kannada or English, respectively. The questionnaire was explained to the girls, and, then, they were asked to fill it. Difficulties faced by the girls in filling the forms were addressed to by the research team. The forms were collected by the team of doctors who were conducting the study. The time duration taken to complete the forms was about 45–60 minutes.

After filling of the questionnaire, a lecture on MHM was delivered to the girls. The lecture covered basic physiology of reproduction, menstrual hygiene management, contraception, and nutrition.

### 2.1. Statistical Analysis

All characteristics were summarized descriptively. For continuous variables, the summary statistics of mean ± standard deviation (SD) were used. For categorical data, the number and percentage were used in the data summaries and diagrammatic presentation. The Chi-square (*χ*^2^) test was used for the association between two categorical variables.

If the *P* value was <0.05, then the results were considered to be statistically significant; otherwise, it was considered as not statistically significant. Data were analyzed using SPSS software v.23.0 and Microsoft office 2010.

## 3. Results

### 3.1. Sociodemographic Characteristics of the Participants

**Figure 1:**
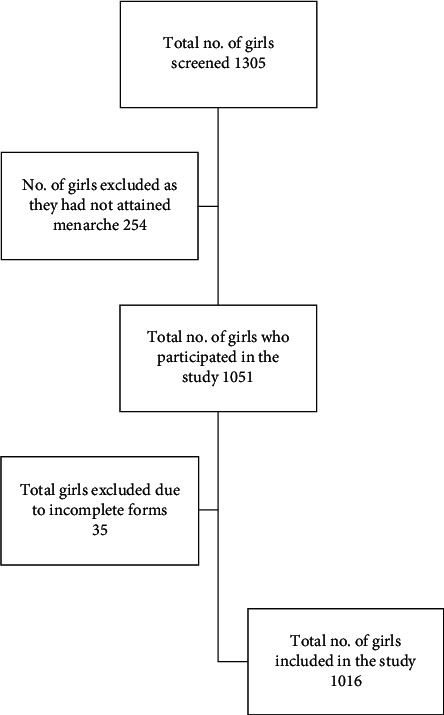
Strobe statement.

In total, there were 1305 girls, of which 1051 had matured. The questionnaire was given to the girls who had matured. A total of 1016 girls were included in the study, and 35 were excluded due to inappropriate filling of the form (Figure 1). A majority of the girls (60.2%) were from the urban area. Most of the girls (56.7%) attained menarche between 12 and 14 years of age. Most of the girls were from families of poor socioeconomic backgrounds with the parents having a poor educational status. Just under half (44.3%) of the fathers of the girls had primary education or were lesser educated. Similarly, 49.7% of the mothers had primary education or were lesser educated. Most of the fathers of the girls were farmers (29.7%) or laborers (21.1%). A majority of the mothers were housewives (69.7%).

### 3.2. Characteristics of the Menstrual Cycle, Menstruation, and Menstrual Morbidities Experienced by the Participants

In the present study, 70.5% of the girls attained menarche by 12 to 14.9 years of age. Majority of the girls had their periods every 28–34 days (37.2%). A significant number of girls (12.2%) of said they have heavy periods. Some of the girls did not wish to disclose details regarding their periods. There was no attempt to coerce them. Most of the girls bled for 6 days or less, however 9% of the girls said that they bleed for 7 or more days (Table 1).

In our study, 62.3% of the girls of the urban area and 61.6% of the girls from the rural area said that they experienced pain during menstruation, and 8.3% of the urban girls and 9.7% of the rural girls said that they required medications for the pain. Other minor ailments such as uneasiness, breast pricking, and leg cramps were also experienced by some of the participants. There was no statistical difference between urban and rural participants with respect to the abovementioned morbidities (Table 2).

### 3.3. Menstrual Hygiene Practices among the Participants

The study observed that 69.8% of the urban girls and 72% of the rural girls were using commercial sanitary napkins whereas 15.4% of the urban girls and 10.1% of the rural girls were using reusable cloths during their periods. There were 14.2% of the urban girls and 16.3% of the rural girls who were using both cloth and pads. Some of the urban girls (12.1%) and rural girls (12.6%) said that they had no knowledge about commercial sanitary pads. A few of the urban girls (7.8%) and rural girls (6.9%) said that said that they were costly. In our study, 14.7% of the urban girls and 15.8% of the rural girls said that they had a problem with disposal of the pad. The reasons given by the girls of both urban and rural areas for using cloth was that the elder women of the house asked them to use cloth and that they were easily available, cheap, and safer than pads. A majority of the urban girls (60%) and the rural girls (51%) who were using cloth as an absorbent were not drying the cloth in sunlight. This is statistically significant (*P*=0.005). Most of the urban girls (59.5%) and rural girls (54.7%) were washing their genitals more than 2 times a day; however, the rest were washing infrequently. It was seen that the rural girls were washing their genitalia less frequently as compared to their urban counterparts (*P*=0.039). The study observed that 92.8% of the urban girls and 94.8% of the rural girls were having bath during menses, and 87.2% were using soap along with water. It was interesting to note that 28.9% of the urban girls and 46.5% of the rural girls disposed their pads by burning them, which is statistically significant (*P* value < 0.001). In addition, 60.3% of the urban girls and 41.38% of the rural girls of then disposed them in the dust bin (*P* < 0.001) and 4.1% of the urban girls and 5.7% of the rural girls buried them. Girls from the rural area were less frequently washing their genitalia and using soap as in comparison to the urban girls, and burning pads was more frequent in the rural girls (*P* < 0.05) (Table 3).

### 3.4. Difficulties Incurred by the Participants at School during Menstruation and MHM at School

School absenteeism was considerable during menstruation with 9% of the urban girls and 8.2% of the rural girls remaining completely absent during periods.

In our study, 18.6% of the urban girls and 17.1% of the rural girls said that they remained absent for a day. It was observed that 52.1% of the urban girls and 54.7% of the rural girls had difficulty in concentration at school, and 10.1% of the urban girls and 12.6% of the rural girls said that they stained their clothes during menstruation.

#### 3.4.1. WASH Facilities at School

In our study, 79.2% of the urban girls and 73% of the rural girls said that they had adequate water and sanitation facilities at school. Facility to change their pads at school was said to be adequate by 21.4% of the urban girls and 23.3% of the rural girls. Most of the urban girls (72.5%) and rural girls (73.8%) said that they could get a spare pad at school. These pads were available with a certain teacher. They were meant for emergency. These pads were not easily available or kept in the washrooms.

Over half of the urban (62.3%) and rural (56.9%) girls said that they could get medication for pain relief at school. The tablets were again usually available with a particular teacher. The study revealed that 36.4% of the urban girls and 33.4% of the rural girls said that they sometimes required help in the school for pain during their periods. In case help was required, teachers and friends were available for help at school and parents at home. The study observed that 26.3% of the urban girls and 23.3% of the rural girls took their teachers help while 39.4% of the urban girls and 38.9% of the rural girls took help of their friends. At home, 40% of the urban girls and 33.7% of the rural girls took their parents help (*P*=0.031), which is statistically significant (Table 4).

### 3.5. Cultural Beliefs Practiced by the Participants

In our study, 46.4% of the urban girls and 40.3% of the rural girls said that they avoided cultural functions during their periods, and 39.4% of the urban girls and 37.6% of the rural girls said that they avoided religious ceremonies and practices during their periods. The number of urban girls and rural girls who were asked to stay away from people during their periods was 18.3% and 17.8%, respectively. It was observed that 8.9% of the urban girls and 10.6% of the rural girls were made to sit outside the house during their periods, which was statistically significant (*P*=0.033). Girls from both the groups were asked to sleep separately from the family members. Restrictions regarding food and water were there in both the groups with 16.7% of the urban and 15.8% of the rural girls not allowed to touch food. Restrictions in eating certain food items were observed in 22.1% of the urban and 20.5% of the rural girls. It was observed that 7.8% of the urban and 8.9% of the rural girls were not allowed to drink water during their periods, 11.6% of the girls coming from rural background were not allowed to drink water from the well as in comparison to 9.5% of the urban girls. Menstruating women were thought to be impure and were not allowed to drink water from the well. A higher percentage of rural girls in comparison to their urban counterparts were made to sit outside the house during their periods (10.6 vs. 6.9%, *P* value 0.033). Girls of both urban and rural background were not allowed to touch anyone during their periods (12.3% vs. 11.95), and 20.3% of the rural girls were not allowed to play during their periods in comparison to 15% of the urban girls which was statistically significant (*P*=0.029) (Table 5).

## 4. Discussion

The study aims at studying the age of menarche, menstrual cycle, menstrual morbidities, menstrual hygiene practices, difficulties incurred by the girls while attending school and WASH facilities at school, and cultural beliefs practiced by the girls of North Karnataka, India. The study was conducted in schools which cater to girls form middle and low socioeconomic background. A majority of the girls in our study attained menarche by 12–14.9 years of age. Various studies conducted in India show that most of the girls attain menarche by 12–14 years in India [9, 10]. Heavy menstrual bleeding (HMB) has been observed in 12.2% of the respondents in our study. Other studies conducted in India show that HMB may be experienced in 4 to 22% of the respondents [3, 9]. Painful Menstruation has been a common symptom experienced by almost 62.3% and 61.6% of the urban and rural girls, respectively. Other aliments of menstruation such as lack of concentration; breast pricking, and leg cramps were also experienced by most of the girls. These symptoms have been documented by other studies as well. In a study conducted in Thiruvananthapuram, Kerala, India, dysmenorrhea stood out to be the commonest menstrual morbidity at 74% followed by back pain, irritability, leg pain, and vulval pain [6]. In another study conducted in Maharashtra, India, 21% of the respondents suffered from dysmenorrhoea [11].

This is very important as the girls may have to endure the pain during the school classes. This may, in turn, cause lack of concentration, discomfort in school, or lead to dropping out of school during menstruation.

Though a majority of the girls in our study were using sanitary napkins, some were using cloth and some were using a combination of both. There was no statistical difference between the urban and rural groups with respect to the nature of absorbent used during menstruation. The use of combined cloth and pad was used more in the rural area (16.3% vs. 14.2%). Other studies in India, Bangladesh, Pakistan, and from the African continent show that cloth is commonly used as an absorbent [9, 12–16]. Usage of cloth in an inappropriate way, without proper washing and drying, can lead to genital infections [4]. A similar number of girls from the urban and rural area were unaware about the use of sanitary napkins. The commonest reason for not using pads was the problem of disposal of the pads. This could be due to lack of sanitary facility at home or at school. Both urban and rural girls felt that the pads were expensive. An almost equal number of rural and urban girls used cloth as an absorbent as it was advised by elders at home. A majority of the girls reported that they did not dry the cloth in sunlight. This was more in the urban girls (60%). Other studies also revealed that the reusable cloths were not been adequately dried in sunlight [7].

In our study, over 90% of both urban and rural girls had a bath during menstruation. Most of them were washing their genitals more than 2 times a day. A majority did use soap to wash. However, it was observed that girls from the rural areas washed less frequently as compared to their urban counterparts. These girls need to be counseled regarding their practices. In a study conducted in Pakistan, 58.2% of the respondents did not take bath during menstruation; however, 80.5% were washing their genitalia during menstruation [16].

It was noticed that only 60.3% of the urban girls and 41.3% of the rural girls could dispose the pad in dustbin. This could be due to lack of sanitary facilities or shyness at home to dispose it in a dustbin. Burning of the pads was more common in the rural girls with 46.5% doing it. Less than 10% of the girls were burying the pads. Similar practices have been noticed in other regions of Karnataka [10].

Over 90% of both the groups of girls went to school during their periods. However, 18.6% of the urban and 17.1% of the rural girls skipped school for a day. There was no significant difference in the groups regarding the school absenteeism. School absenteeism has been observed in studies conducted in Africa and in South Asian countries [17, 18]. Over half of the girls in both groups showed lack of concentration during the periods, and 10.1% of the urban and 12.6% of the girls said that they had staining of the clothes during periods. This may be due to infrequent changing of the pad. The reason for infrequent changing could again be inadequate WASH facilities or shyness of the girls to change the sanitary pad at school. The toilet facilities were present at school in most of the cases; however, 79.2% of the urban and 73% of the rural girls said that the water supply was inadequate, pointing to the poor functioning of the toilets. In Asia, according to the WHO/UNICEF Joint monitoring program for Water Supply, Sanitation, and Hygiene (WASH), 50–75% of the schools had adequate sanitary facility [19]. Over 70% of the girls said that sanitary napkins were available at the school, but not easily accessible, as only a few teachers had them, and the girls were expected to contact a particular teacher which may not be feasible at times. Pain relief was available at schools, in the form of tablets, which were again not easily available. There were no vending machines or extra sanitary napkins placed in the toilets.

Many of the girls were practicing cultural restrictions during the periods, they were abstaining from religious and cultural ceremonies, avoiding certain foods and water, sitting outside the house, and refraining from touching people, and some of them were not allowed to play and were asked to sleep away from the family. Some rural girls were made to sit outside the house and were not allowed to play during periods as in comparison to the urban girls. Such practices have been seen in various religions in India. Cultural restrictions have also been seen in a study conducted in Jodhpur, Rajasthan, India, in which 49.4% of the urban girls and 30.7% of the rural girls practiced cultural restrictions. Interestingly, a significant number of girls said that restrictions should be imposed [13]. In a study conducted in West Bengal, 64.72% of the urban girls and 78.58% of the girls from rural area practiced cultural restrictions during menstruation [20].

In most of the Indian houses, women are restricted from touching sour things such as pickles or restricted from their consumption. Women are restricted to enter holy places, which is followed by different religions as they will bring impurity. In some customs, the first menses or menarche of a girl is celebrated as per their culture, but at the same time, women who are menstruating are asked not to partake in any rituals as they are considered filthy and impure [8].

## 5. Conclusions

Menstruation, menstrual morbidities, and menstrual hygiene management are issues which are required to be adequately addressed. Inadequate facilities at school tend to cause school absenteeism. Openness to the topic and ground level recognition of the deficiencies at schools should reduce the school absenteeism. Cultural beliefs practiced also reduce the schooling days of the girl. This further leads to reduced academic performance. Counseling regarding such matters is required. A realistic approach by schools regarding MHM and WASH facilities is required. Also, we would recommend that Menstrual Hygiene Management should be included in the curriculum of schools and a dedicated approach to the issue is required to bring out change especially in girls from underprivileged families.

## Figures and Tables

**Table 1:** Characteristics of the menstrual cycle and menstruation in the participants.

Age of menarche in years	Frequency	Percent
7.0-7.9	1	0.1
8.0-8.9	0	0
9.0-9.9	0	0
10.0-10.9	5	0.5
11.0-11.9	41	4
12.0-12.9	143	14.1
13.0-13.9	255	25.1
14.0-14.9	321	31.6
15.0-15.9	196	19.3
16.0-16.9	51	5
17.0-17.9	1	0.1
18.0-18.9	2	0.2
Duration of bleeding during periods		
<21 days	118	11.6
Every 21–27 days	227	22.3
Every 28–34 days	378	37.2
>35 days	221	21.8
Not disclosed	72	7.1
Amount of bleeding during periods as perceived by the participants		
Normal	766	75.4
Heavy	124	12.2
Scanty	100	9.8
Not disclosed	26	2.6
Length of the menstrual cycle		
<4 days	414	40.7
5–6 days	473	46.6
>7 days	91	9
Not disclosed	38	3.7

**Table 2:** Menstrual morbidities experienced by the participants by the place of residence.

Menstrual morbidities	Urban		Rural		*P* value
	*N*	Percentage	*N*	Percentage	
Pain during periods	381	62.3	249	61.6	0.842
Drugs used during periods	51	8.3	39	9.7	0.469
Complaint during menses					
Uneasiness	171	27.9	126	31.2	0.265
Depression	186	30.4	135	33.4	0.310
Leg cramps	193	31.5	123	30.4	0.713
Breast pricking	24	3.9	14	3.5	0.708

*N* is the number of participants.

**Table 3:** Menstrual hygiene practices among the participants by the place of residence.

Menstrual hygiene practices	Urban		Rural		*P* value
	*N*	Percentage	*N*	Percentage	
Materials used during periods					
Pad	427	69.8	291	72.0	0.055
Cloth	96	15.4	41	10.1	
Pad and cloth	87	14.2	66	16.3	
Not disclosed	5	0.7	6	1.5	
Reason for not using pad					
No knowledge	74	12.1	51	12.6	0.800
High cost	48	7.8	28	6.9	0.588
Shyness	27	4.4	11	2.7	0.165
Disposal problem	90	14.7	64	15.8	0.621
Unavailability	19	3.1	17	4.2	0.352
Reason for using cloth					
Easy availability	57	9.3	40	9.9	0.755
Low cost	18	2.9	9	2.2	0.489
Reusable	24	3.9	19	4.7	0.545
Safer than pad	72	11.8	42	10.4	0.499
Advised by elders	87	14.2	59	14.6	0.863
Place of drying cloth					
Sunlight	245	40.0	198	49.0	0.005^*∗*^
Without sunlight	367	60.0	206	51.0	
Cleaning of genitals					
>2 times	364	59.5	221	54.7	0.039^*∗*^
<2 times	189	30.9	153	37.9	
Only during bathing	40	6.5	15	3.7	
Not disclosed	19	3.1	15	3.7	
Materials used for washing					
Water and soap	550	89.9	341	84.4	0.009^*∗*^
Water	62	10.1	63	15.6	
Bathing during menses					
Yes	568	92.8	383	94.8	0.204
No	44	7.2	21	5.2	
Disposal of pad					
Burning	177	28.9	188	46.5	<0.001^*∗*^
Dustbin	369	60.3	167	41.3	<0.001^*∗*^
Burying	25	4.1	23	5.7	0.237

*Note.* ^*∗*^Significant at 5% level of significance (*P* < 0.05); *N* is the number of participants.

**Table 4:** Difficulties incurred by the participants at school during menstruation by the place of residence.

Difficulties incurred	Urban		Rural		*P* value
	*N*	Percentage	*N*	Percentage	
Going to school during periods					
Yes	557	91.0	371	91.8	0.650
No	55	9.0	33	8.2	
Number of days absent from school during periods					
0	417	68.1	286	70.8	0.111
1	114	18.6	69	17.1	
2	31	5.1	12	3.0	
3	16	2.6	5	1.2	
4	24	3.9	18	4.5	
5	10	1.6	14	3.5	
Problems faced					
Concentration during periods	319	52.1	221	54.7	0.420
Staining of clothes	62	10.1	51	12.6	0.216
Change of pad facilities at school	131	21.4	94	23.3	0.484
Adequate water in toilet at school	485	79.2	295	73.0	0.021^*∗*^
Pads available at school	444	72.5	298	73.8	0.670
Pain relief tablets at school	381	62.3	230	56.9	0.090
Help for pain at school	223	36.4	135	33.4	0.324
Other problem	392	64.1	215	53.2	0.001^*∗*^
Help from					
Teacher	161	26.3	94	23.3	0.274
Friends	241	39.4	157	38.9	0.869
Parents	247	40.4	136	33.7	0.031^*∗*^

*Note.* ^*∗*^Significant at 5% level of significance (*P* < 0.05); *N* is the number of participants.

**Table 5:** Cultural beliefs practiced by the participants by the place of residence.

Cultural beliefs	Urban		Rural		*P* value
	*N*	Percentage (%)	*N*	Percentage (%)	
Avoiding cultural functions	284	46.4	163	40.3	0.057
Avoiding religious functions	241	39.4	152	37.6	0.574
Stay away from people	112	18.3	72	17.8	0.846
Not touching food	102	16.7	64	15.8	0.728
Sleeping away from people	82	13.4	59	14.6	0.587
Sitting outside the house	42	6.9	43	10.6	0.033^*∗*^
Not to touch anyone	75	12.3	48	11.9	0.858
Not to play	92	15.0	82	20.3	0.029^*∗*^
Not to eat something	135	22.1	83	20.5	0.565
Not to drink water	48	7.8	36	8.9	0.545
Not to drink well water	58	9.5	47	11.6	0.269

*Note.* ^*∗*^Significant at 5% level of significance (*P* < 0.05); *N* is the number of participants.

## Data Availability

The data used to support the findings of this study are included within the article. Additional data can be obtained on reasonable request to the corresponding author by e-mail.
